# Invisible Erosion of Human Capital: The Impact of Emotional Blackmail and Emotional Intelligence on Nurses’ Job Satisfaction and Turnover Intention

**DOI:** 10.3390/bs13010037

**Published:** 2022-12-31

**Authors:** Wei-Yuan Lo, Yu-Kai Lin, Chun-Yu Lin, Hsiang-Ming Lee

**Affiliations:** 1 Department of Business Administration, National Taipei University, New Taipei City 237, Taiwan; 2 Department of Health and Welfare, University of Taipei, Taipei 111, Taiwan; 3 Department of Business Administration, National Taipei University of Business, Taipei 100, Taiwan

**Keywords:** emotional blackmail, emotional intelligence, job satisfaction, turnover intention, nurses

## Abstract

Emotion is a compelling factor in the retention and job satisfaction of professionals, and the impacts of emotional feelings and reactions have become an indispensable issue in the nursing workforce. Drawing on the cognitive theory of emotions, this study bridges the research gap to investigate the relationships among emotional blackmail, emotional intelligence, job satisfaction, and turnover intentions of nurses. A cross-sectional design was used to collect data from a sample of 374 full-time nurses in Northern Taiwan. Hypotheses were tested and analyzed by means of SPSS 22, structural equation modeling (SEM), and PROCESS v3.3. The results revealed the direct relationships between emotional blackmail, job satisfaction, and turnover intentions, and job satisfaction partially mediated the relationship between emotional blackmail and turnover intentions. However, there is no statistical support that emotional intelligence moderates the relationships between emotional blackmail and job satisfaction and turnover intentions. Healthcare organizations must take the initiative and form strategies that will help balance nurses’ work stresses. These strategies should aim to reduce unnecessary demands from supervisors, patients, and co-workers, as well as in the socio-emotional domain.

## 1. Introduction

Nurses as a sustainable human resource is an emerging and compelling issue [1]. Nearly half of the World Health Organization (WHO) member countries reported that their hospitals average fewer than 3 nurses per 1000 residents, and even more alarming is that more than a quarter (27%) of these member countries report less than 1 nurse per 1000 residents [2]. Accordingly, excessive turnover of nurses results in a loss of competent employees, which incurs extra costs for training and recruiting new nurses [3] and concomitantly results in inefficient hospitals [4] that produce less reliable levels of organizational performance and patient safety due to medical errors [5].

Previous research has demonstrated that healthcare jobs are inherently stressful professions that are associated with long work hours, difficult work conditions, complicated and numerous occupational health cases, and interpersonal contagious hazards [1]. However, healthcare systems such as hospitals are increasingly required to be more patient-oriented, which means that healthcare employees must be more responsive and interactive in the workplace [6]. Governments, healthcare industries, and the public now tend to view the nursing profession as a type of personal health service [7,8]. In order to enhance patients’ satisfaction, supervisors and colleagues in hospitals often make unreasonable demands on nursing staff [8]. Thus, the impact of the emotional feelings and reactions of incumbent healthcare workers has become an indispensable issue [9]. Previous studies have noted that emotional responses often influence customers’ evaluations and interactions with high-contact services provided by frontline employees that include medical and other professional services [6].

Emotion is an affective variable and is considered a complicated collection of the regulatory roles of behavior [10]. Academics have extensively discussed and empirically proved that emotional issues such as psychological conditions and interpersonal relationships in the healthcare environment of nurses are antecedents of job satisfaction, turnover intentions, and actual turnover [11,12,13]. Drawing on the cognitive theory of emotions, Solomon [14] illustrated that emotion is a kind of cognitional response to the situation and will trigger a series of rational and purposive actions. With emotional judgment, when the individual feels heavy inner pressure, he/she will consider leaving [15]. Individuals develop psychological reactions relative to changes in their environment. The nature of psychological conditions and interpersonal relationships can become a critical aspect that can negatively affect a nurse’s job satisfaction when interpersonal tension is increased, which can reduce productivity and enthusiasm and trigger them to leave [15,16]. Based on this argument, the nature of the psychological condition and interpersonal relationships can become critical aspects that can negatively affect a nurse’s job satisfaction when interpersonal tension is increased, which can reduce enthusiasm and trigger them to leave [15].

It is not uncommon for nursing staff to endure inappropriate emotional behavior, such as unreasonable demands in the workplace [13]. Past research has extensively discussed and empirically proven that emotional issues, such as bullying and abuse, in the healthcare environment of nurses, are antecedents of job satisfaction, turnover intentions, and actual turnover [11,12,13]. However, to our best knowledge, no empirical research has examined the impact of nurses’ perceptions of emotional blackmail at work. Emotional blackmail is defined as a powerful but invisible tool that is used to force a person to do something [17]. Liu [16] has demonstrated that emotional blackmail is a kind of stress, threat, and assigning blame from others. In a collectivist society such as Taiwan, individuals are more eager to be the in-groups of the organization by demonstrating cooperative work attitudes and behaviors, and thus, tend to hide their true feelings and reactions under the circumstance of unreasonable emotional demands [18]. In order to identify and fill the gaps in the literature, the first aim of the present study focuses on the impact of emotional blackmail on the turnover intentions and job satisfaction of nursing staff.

Nurses have to face numerous emotional demands and interpersonal interactions with the public under intense situations, which makes it likely they will be exposed to negative emotions [13]. These situations require them to regulate their feelings and expressions. Hence, the self-regulation of emotions has become an important management issue [19]. Emotional intelligence refers to the ability to perceive and manage emotional messages and information, perceive individuals’ and others’ emotions, and the ability to understand the complexities of emotional transmission [20]. Previous research has argued that emotional intelligence can effectively reduce negative stress responses among superiors, subordinates, and colleagues [19,21] and can affect interpersonal interactions [19,22,23].

Emotional intelligence can effectively reduce negative stress responses among superiors, subordinates, and colleagues [19,21] and can affect interpersonal interactions [22,23]. Individuals with higher emotional intelligence will have a higher ability to dominate their interactions with others [24]. Prior studies have also demonstrated that individuals with a high level of emotional intelligence can have positive effects on their attitudes [22] and reduce their negative responses among co-workers [19], and in turn, decrease their turnover intentions [12,13]. Healthcare organizations are highly complicated and professional settings that require tight interpersonal interactions [25]. Researchers have empirically established that emotional intelligence affects the work outcomes, such as job satisfaction, turnover intentions, and actual turnover, of nursing staff [26]. To anchor the theoretical arguments about relative research, this study further examines the moderating effect of emotional intelligence on the relationship between emotional blackmail, turnover intentions, and job satisfaction.

Past studies of emotional issues, such as bullying, in the healthcare environment of nurses are antecedents of job satisfaction, turnover intentions, and actual turnover [11,12,13]; we follow calls to extend the research of emotional issues by testing our theoretical model and offer three contributions to the literature in the following aspects. First, we draw on the cognitive theory of emotions and adopt a cognitional insight to explicate the relationships between emotional blackmail, turnover intentions, and job satisfaction proposed in the study and complement emotional blackmail as a distinct perspective to facilitate a more holistic understanding of emotional issues in healthcare organizations. Accordingly, the current research further investigates the mediating role of job satisfaction between emotional blackmail and turnover intentions. Thus, it helps demonstrate a fuller picture of the quality of interpersonal interactions, which will arouse employees’ job satisfaction and, in turn, reduce their intentions to leave. Finally, an individual’s emotional intelligence is expected to accurately transfer valid information to interact with others [27]. Thus, we further examine the moderating effect of emotional intelligence on the relationships between emotional blackmail, job satisfaction, and turnover intentions to enrich the academic fields.

The remainder of this paper is organized as follows: the next section presents the literature review of the four constructs and postulates the relevant hypotheses based on the results of previous studies. Section 2 demonstrates the research methodology and the data collection used in this study. Section 3 examines the results of the hypotheses analyses. Section 4 discusses the theoretical and practical implications of the findings, the limitations of the study, and future research directions.

### 1.1. Literature Review and Hypothesis Development

#### 1.1.1. Theoretical Framework

Based on the cognitive theory of emotions, the emotional state is determined by cognitive factors [28]. Individuals develop psychological reactions relative to changes in their working environment. In this model, negative reactions become a psychological response to the negative aspects of psychological working conditions and interpersonal relationships, such as lack of support from organizational leaders and co-workers, which have been accurate predictors when employees consider leaving their jobs [1].

The nature of psychological conditions and interpersonal relationships can become a critical aspect that can negatively affect an employee’s job satisfaction when interpersonal tension is increased, which can reduce productivity and enthusiasm and trigger them to leave [15,16]. Emotional blackmail is defined as a powerful but invisible tool that is used to force a person to do something [17]. The emotional and mental health conditions of nurses could deteriorate because of excessive work demands and a lack of support, which could then develop into withdrawal behaviors [29].

#### 1.1.2. The Impact of Emotional Blackmail on Turnover Intentions

Employees normally make efforts to assimilate into organizations by maintaining good inter-relationships and avoiding conflicts with others [30]. Emotion, therefore, becomes a vulnerable affect to be manipulated [31]. Emotional blackmail occurs when individuals are emotionally stressed by others, which generates intense and conflicting emotions [15]. Simply put, when emotional blackmail happens, one party manipulates others in order to achieve his/her purposes, including threats, posting negative labels, brawling and yelling, shaping a pitiful image, mocking words, and ignoring, all of which could result in disturbing situations that harm the health and well-being of others [17].

Nurses in hospitals inevitably suffer irrational demands, such as emotional blackmail, in interpersonal relationships when interacting with supervisors, colleagues, and the public. According to the cognitive theory of emotions, emotions are made up of a series of rational and purposeful actions that lead to emotional judgments and reactions [32]. In this model, turnover intentions become a psychological response to the negative aspects of working conditions [32]. Previous studies have found that the emotional blackmail of civil servants is positively related to turnover intentions [15]. More specifically, when individuals perceive emotional blackmail, they are more likely to leave as a result of the emotional pressure [33]. Therefore, we hypothesized that:

**H1:**  *Emotional blackmail is positively associated with turnover intentions.*

#### 1.1.3. The Impact of Emotional Blackmail on Job Satisfaction

Job satisfaction is an employee’s emotional reaction or self-appraisal of their own job experience and is determined by an employee’s state of mind concerning their work environment and perceived level of pressure [2]. Job satisfaction is usually determined by an employee’s state of mind concerning their work environment and perceived level of pressure [2]. Previous studies have established employees’ job satisfaction via facets such as the perceived quality of the relationship with supervisors and co-workers and work conditions [19]. Studies on workplace emotional blackmail have indicated that both the internal and external stimuli from emotions can affect an employee’s physical, psychological, cognitive, and behavioral reactions [32,34]. Liu [16] has revealed emotional blackmail of employees from fifteen companies could predict a variety of work outcomes, such as job satisfaction and well-being. Accordingly, unpleasant work environment factors such as emotional blackmail could affect a nurse’s motivation to work and impact their level of job satisfaction. In this study, we hypothesized that:

**H2:**  *Emotional blackmail is negatively associated with job satisfaction.*

#### 1.1.4. The Relationship between Emotional Blackmail, Job Satisfaction, and Turnover Intentions

The relationship between job satisfaction and turnover intentions is well established in the literature [35], and relevant empirical studies have found that job satisfaction is a major factor affecting individuals’ withdrawal attitudes [35,36]. In healthcare institutions, job satisfaction is a primary predictor of nurses’ turnover intentions in different countries and contexts [11]. In medical institutions, job satisfaction has been identified as negatively related to nurses’ turnover intentions [37,38]. Accordingly, we hypothesized that:

**H3:**  *Job satisfaction is negatively associated with turnover intentions.*

Emotion is an important part of the human psychological state and a motivator of reactions and behaviors [39]. Negative emotional conditions may have negative effects on job satisfaction, and in turn, on turnover intentions [39]. Alotaibi et al. [40] have demonstrated that a stressful work environment had impacted the job satisfaction of Saudi nurses and prompted their intentions to leave the profession. In Taiwan, Lo et al. [41] have indicated that full-time hospital nurses tended to leave due to the impact of high levels of job stress and low levels of job satisfaction. Based on the abovementioned studies, one could postulate that when nurses perceive emotional blackmail, it reduces their level of job satisfaction and increases their intentions to leave. In this study, we postulated that:

**H4:**  *Job satisfaction mediates the relationship between emotional blackmail and turnover intentions.*

#### 1.1.5. The Impact of Emotional Intelligence on Emotional Blackmail, Job Satisfaction, and Turnover Intentions

Proponents have argued that emotional intelligence is a concept that is distinct from personality traits and mental ability [22]. Emotional intelligence refers to “the ability to appraise one’s own and others’ emotions and to utilize this message to guide actions” [42]. Emotional intelligence, therefore, is an individual’s capacity to enhance cognitive activities [42], which can be a positive predictor of work outcomes in the workplace [22,43]. Employees with high emotional intelligence experience lower levels of work stress and avoid negative behaviors when their work-related stress rises [44]. Further, individuals with high levels of emotional intelligence not only possess the ability to regulate their own emotions but those of others to achieve their purposes [19]. This may explain why emotionally intelligent employees exhibit fewer intentions to leave despite unfavorable working conditions. The crucial role of emotional intelligence has also been recognized in cases of nursing staff working in healthcare organizations [45]. As a result, we hypothesized that:

**H5:**  *Emotional intelligence moderates the relationship between emotional blackmail and turnover intentions.*

Emotional intelligence affects a wide array of work behaviors [44] and can also predict crucial work-related outcomes [19]. Previous studies on emotional intelligence and workplace outcomes have indicated that emotional intelligence is related to job performance, job satisfaction, and productivity [19,20,46]. Employees with a higher level of emotional intelligence are more likely to experience positive psychological reactions to factors such as job satisfaction. Sy et al. [43] have demonstrated a positive relationship between emotional intelligence and job satisfaction. Employees who have higher emotional intelligence are better at appraising and regulating their own feelings as well as those of others in the workplace, and they tend to experience more positive moods and emotions and are generally more satisfied with their jobs [43]. 

The positive relationship between emotional intelligence and job satisfaction is also supported in research on nursing [47]. Nurses who have higher emotional intelligence tend to report higher satisfaction. Nurses must deal with events bound with emotions, such as illness and death. Thus, nursing staff has to manage stressful situations imposed by the work environment, and at the same time, they are obliged to perform in the most effective way. Therefore, it is inferred that no matter what kind of work stress employees are facing, employees with high emotional intelligence are less inclined to reduce their job satisfaction. In other words, a higher level of emotional intelligence equates to more positive psychological reactions to factors such as job satisfaction. Based on the abovementioned facts and H4, we postulated that:

**H6:**  *Emotional intelligence moderates the relationship between emotional blackmail and job satisfaction.*

**H7:**  *The indirect effect of emotional blackmail on turnover intentions through job satisfaction moderated by emotional intelligence.*

**Figure 1:**
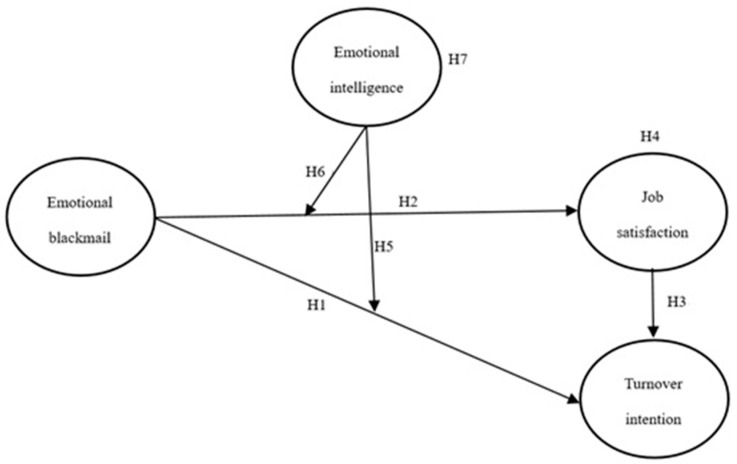
Research framework.

The hypotheses are summarized in Table 1, and the framework of this research is shown in Figure 1.

## 2. Methods

### 2.1. Research Design

This study employed a quantitative and self-rating research design in a public hospital with 401 full-time nurses in Northern Taiwan. A purposive sampling and hospital-based study design were used to collect data. With ethical approval from the Hospital Research Ethics Committee, we visited the nursing stations of each ward and explained the content of the project and the purposes of the questionnaire to the nursing staff, and then distributed the anonymous questionnaire to the nursing staff to fill out during May to June 2019. Nurses voluntarily returned their questionnaires into a collection box in the nursery room after they had completed their questionnaires. The directors of the nursing department assisted with the retrieval of the questionnaires from each collection box in the nursery rooms after nurses completed and returned the questionnaires.

### 2.2. Research Participants and Procedures

If a questionnaire was incomplete or the reverse items were missed, the questionnaire was deemed invalid and eliminated. With 401 distributed, 374 valid responses were returned for a response rate of 93.27%. Most of the respondents were female (96.3%), and the vast majority of respondents were 20–29 years of age (37.7%). Slightly less than half of the respondents had more than 9 years of work experience (45.5%). More details of the respondent profiles are provided in Table 2.

### 2.3. Data Analysis

The data for this research were accessed from a cross-sectional survey. With SPSS 22, we used the means of each variable to examine the standard deviance, correlations, discriminant validity, common method variance (CMV), and the results of direct effects. Structural Equation Models (SEM) is a statistical technique of multivariate analysis allowing researchers to test and estimate causal relationships simultaneously based on statistical data and research constructs and questions [48,49]. In addition to answering a set of interrelated in a single, systematic, and comprehensive analysis, we employed SmartPLS version 4 to examine the discriminant validity assessment through heterotrait–monotrait (HTMT) ratio, applied AMOS 21 to conduct confirmatory factor analysis (CFA), and employed PROCESS v3.3 for SPSS to examine the mediating, moderating, and moderated mediating effects.

### 2.4. Measures

We employed a quantitative and self-rating research design. The standard back-translation procedure was followed to mitigate the translation from English to Chinese [50]. We also consulted 6 experts, 3 scholars, and 3 practical nurses to ensure the questionnaire was suitable for use with Taiwanese nurses. All responses were given on a 7-point Likert-type scale; response options ranged from 1 ‘totally disagree’ to 7 ‘totally agree’.

Emotional blackmail. A 24-item scale developed and validated by Liu and Jhuang [51] was used to measure the nurses’ emotional blackmail. Emotional blackmail may come from sources such as supervisors, colleagues, subordinates, and customers and may take on different cognitive patterns [52]. Thus, all sources of nurses’ emotional blackmail in the workplace were noted. A sample item was, ‘The supervisor will humiliate me with inappropriate words.’ (see Appendix A). The Cronbach’s alpha was 0.95 in this study.

Emotional intelligence. Emotional intelligence as a set of interrelated skills consists of four dimensions, including the capabilities of self-emotions appraisal, others-emotions appraisal, use of emotion, and regulation of emotion [44]. A 16-item scale developed and validated by Law et al. [44] was used to measure emotional intelligence. The measure combines the four dimensions of emotional intelligence. A sample item was, ‘I have a good sense of why I have certain feelings most of the time.’ (see Appendix A). The Cronbach’s alpha was 0.92 in this study.

Job satisfaction. A 3-item scale of “The ‘Michigan Organizational Assessment Questionnaire” developed and validated by Cammann et al. [53] was used to assess nurses’ job satisfaction. A sample item was, ‘In general, I like working here.’ (see Appendix A). The Cronbach’s alpha was 0.92 in this study.

Turnover intention. We adopted the measurement of “Turnover Intention” that was established by Kelloway et al. [54] to assess turnover intentions. A sample item was, ‘I am actively thinking about leaving this organization.’ (see Appendix A). The Cronbach’s alpha was 0.91 in this study.

## 3. Results

### 3.1. Inter-Correlations of Variables, Reliability, and Validity

Table 3 presents the means, standard deviations, composite reliability (CR), the average variance extracted (AVE), and correlations of the variables. All of the correlation coefficients between variables had medium, or lower, correlations. The composite reliability (CR) values ranged from 0.947 to 0.972 in this study and were all above the recommended minimum values of 0.70 [55], and the AVE of each construct exceeded the recommended value of 0.50 [55]. According to Hair et al. [56], the estimated inter-correlations among the variables were less than the square roots of AVE for each variable, which supports the discriminant validity of the scales. We further employed the heterotrait–monotrait (HTMT) as a criterion to examine the discriminant validity. As shown in Table 4, the values ranged from 0.308 to 0.568 in this study and were all below the threshold of 0.85, which also supports the discriminant validity of the scales [57,58,59].

### 3.2. Common Method Variance

Both ex-ante and ex-post remedies were implemented to minimize response errors and examine common method variance (CMV). Respondents were informed all their responses were voluntary, confidential, and anonymous. They were assured that there were no right or wrong answers in order to help them minimize response bias. Harman’s one-factor test was used to examine the effects of common method variance in posthoc testing [60]. The first principal factor explains less than 31% of the variance (<50%), which suggested that there were no serious CMV problems. Additionally, all variance inflation factors (VIF) were below 2.0 (<10), which means there were no issues with multi-colinearity [61].

### 3.3. Model-Data Fit

We performed confirmatory factor analyses (CFA) to evaluate the fit of our data to this measurement model. The RMSEA was 0.09, which was a mediocre fit, and the χ2 = 283.305, CMIN/*df* = 3.99, RMSEA = 0.09, SRMR = 0.06, CFI = 0.933, TLI = 0.914, IFI = 0.933, GFI = 0.905. Thus, the goodness-of-fit indexes for the measurement model were acceptable [56,62].

### 3.4. Direct, Indirect, Mediating, Moderating, and Moderated Mediating Results

Table 5 summarizes the regression results. Models 2 and 5 revealed emotional blackmail was significantly and positively related to turnover intentions (Model 2, β = 0.488, *p* < 0.001), and the negative relationship to job satisfaction was significant (Model 5, β = −0.379, *p* < 0.001). Model 3 revealed that the negative effect that job satisfaction exerted on turnover intentions also was significant (β = −0.398, *p* < 0.001). Accordingly, H1–H3 were supported.

Model 8 of PROCESS v3.3 for SPSS was used to test the proposed mediating, moderating, and moderated mediating hypotheses [63]. There were 2000 bootstrap samples obtained, and when no confidence interval contains 0, the effect is supported [63]. As for the indirect and moderated effects, according to Table 6, the mediating effect of job satisfaction showed no confidence interval at 0, which indicates that H4 was supported. H6 and H7, however, were not supported.

**Figure 2:**
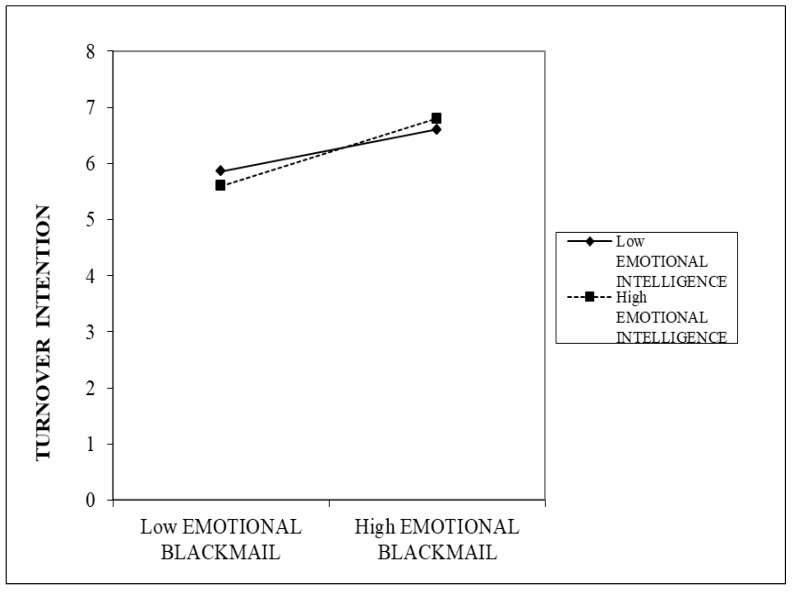
Moderating effect of emotional intelligence on the relationship between emotional blackmail and turnover intentions.

In Table 6, the moderating effect on turnover intentions did not include 0, which indicated H5 was significant. Following the conclusions of Cohen and Cohen [64], however, we examined the high and low values as plus and minus one standard deviation from the mean. The interaction is graphically displayed in Figure 2. The plots of the interaction terms showed that when there was a high level of emotional intelligence, the relationship between emotional blackmail and turnover intentions was stronger than the lower level. As a result, H5 was not supported. The results of the hypotheses are summarized in Table 7.

## 4. Discussion and Conclusions

### 4.1. Discussion

The irrational emotional demands in the workplace could be the critical but invisible factor to influence the attitudes of nurses. Based on the cognitive theory of emotions, this research examined the effects of emotional blackmail on the turnover intentions and job satisfaction of nursing staff, as well as the mediating role of job satisfaction on the relationship between emotional blackmail and turnover intentions. Using data collected from a public regional hospital in Northern Taiwan, the results of statistical analyses indicated that the perception of nurses’ emotional blackmail is positively associated with nurses’ turnover intentions (H1) and negatively associated with job satisfaction (H2). Moreover, job satisfaction is negatively associated with nurses’ turnover intentions (H3) and partially mediates the relationship between emotional blackmail and turnover intentions (H4). 

Previous studies have established employees’ psychological working conditions and the perceived quality of their interpersonal relationships have been accurate predictors of their job satisfaction, turnover intentions, and actual turnover [1,19]. Furthermore, visible negative emotional conditions, such as stress, bullying, and abuse, may have negative effects on job satisfaction, and in turn, on turnover intentions [39]. The emotional and mental health conditions of nurses can deteriorate because of excessive work demands and a lack of support, which can then develop into turnover intentions [29]. In accordance with relevant empirical studies in healthcare institutions, the results of this study significantly advance previous research by investigating the field that emotional blackmail of nurses in the workplace has indicated that both the internal and external stimuli from emotions can affect an employee’s physical, psychological, cognitive, and behavioral reactions, and could predict a variety of work outcomes such as nurses’ job satisfaction and turnover intentions.

This research further developed and examined a moderated mediation model that posits job satisfaction as a mediator between emotional blackmail and turnover intentions and emotional intelligence as a moderator to such mediation. Findings from this research exhibited that nurses’ emotional intelligence was not found to be a moderator to the relationship between emotional blackmail and job satisfaction (H6), and the moderated mediating effect was not supported (H7). Moreover, the result of statistical analysis indicated that the moderating effect of emotional intelligence on the relationship between emotional blackmail and turnover intentions was significant. However, we examined the high and low values as plus and minus one standard deviation from the mean. The plots of the interaction terms showed that when there was a higher level of emotional intelligence, the relationship between emotional blackmail and turnover intentions was stronger than the lower level. Conversely, H5 was not supported.

Previous research has demonstrated that employees with higher emotional intelligence would experience lower levels of work stress and avoid negative behaviors when their work-related stress rises [44]. Employees who have higher emotional intelligence are better at appraising and regulating their own feelings as well as those of others in the workplace, and they tend to experience more positive moods and emotions and are generally more satisfied with their jobs [43]. As a result, emotionally intelligent employees will exhibit fewer intentions to leave despite unfavorable working conditions. However, the results of this study revealed that emotional intelligence failed to moderate the relationship between emotional blackmail, turnover intentions, and job satisfaction. An interesting finding is that, according to the result of statistical analysis, the effect of emotional intelligence even conversely increases the turnover intentions of nurses in medical institutions. As such, the findings of the study provide some further problem awareness in explaining the transmission mechanism of the effects of emotional intelligence in healthcare organizations.

### 4.2. Theoretical Implications

Nurses are important and sustainable assets in healthcare systems, and the turnover rate of nurses is recognized as a compelling and multifaceted issue [65]. In hospitals, nurses confront various stressful and dissatisfying situations, such as heavy workloads, patients with excessive demands, and conflicting job demands [13]. The empirical research of Gkorezis et al. [38], Alotaibi et al. [40], and Lo et al. [41] has indicated that negatively emotional conditions positively relate to nurses’ turnover intentions and negatively relate to their job satisfaction. Our findings are in line with relevant studies and reiterate and extend the importance of emotional issues in the workplace.

In this study, we also examined the moderating role of emotional intelligence. Previous researchers found that higher emotional intelligence regulates employees’ emotions and allows them to deal with negative effects such as stress from emotional blackmail. Further, higher emotional intelligence often dominates the ability to perceive, appraise, and manage emotions, which often enhances job performance [19]. Hence, nurses with higher emotional intelligence are generally able to respond positively to irrational demands of the workplace and actually achieve better job satisfaction with fewer turnover intentions. In contrast, we did not find statistical support that emotional intelligence moderates the relationship between emotional blackmail and job satisfaction, as well as the relationship between emotional blackmail and turnover intention.

Based on the abovementioned contributions, the research also has the following theoretical implications. First, in the literature, while sufficient results reveal that emotional issues are associated with employees’ turnover intentions and job satisfaction [11,15,38], little research has demonstrated how emotional blackmail could have an influence on these outcomes. In addition to visible and predictable irrational demands such as stress, threats, abuse, and bullying, this study sheds light on and enriches the research field to investigate the invisible factor, emotional blackmail, which would erode the human capital without warning. According to our findings, the study is based on the cognitive theory of emotions and explains that nurses’ perceptions about emotional blackmail have a positive impact on turnover intentions and a negative impact on job satisfaction, and, further, job satisfaction played a partial mediating role between emotional blackmail and turnover intentions. More specifically, when nurses perceived suffering from irrational emotional demands in their workplace, they were less satisfied with their jobs and were more likely to leave their hospital.

Second, most research on the moderating role of emotional intelligence has demonstrated that individuals with a higher level of emotional intelligence would have more ability to cope with stress yielding in the organization [19]. On the contrary, we did not find that the emotional intelligence of nurses in a public hospital in Northern Taiwan has moderating effects on the relationship between emotional blackmail and turnover intention and job satisfaction. Our research findings provide an avenue that emotional blackmail is a kind of powerful but invisible tool that is used to force a person to do something [17]. Thus, the obedience of nurses would be the surface acting by the regulating ability of emotional intelligence, which would not enhance their job satisfaction, and in turn, decrease their turnover intentions.

Third, an interesting finding is that along with increases in irrational emotional and work demands, such as emotional blackmail, hospital nurses with higher emotional intelligence had higher levels of turnover intentions. This could suggest that nurses with higher emotional intelligence may be more eager to be the in-groups of the organization by demonstrating cooperative work attitudes and behaviors, which is often seen in the collectivist culture of Taiwan. Thus, people tend to hide their true feelings [18,66,67]. Accordingly, once nurses with higher emotional intelligence perceived increased suffering from emotional blackmail and felt a sense of social exclusion, they had a tendency to leave. In addition, job demands are higher for nurses in healthcare systems, and there are alternative employment opportunities for them. Therefore, nurses who perceive higher levels of emotional blackmail can certainly leave disadvantageous jobs before suffering from more mental and physical abuse.

### 4.3. Implications for Practice

Healthcare organizations are highly complicated and professional systems that require professionals from different departments to work together [25]. Nursing managers should develop and implement interventions from psychological, professional, and environmental perspectives in order to enhance the job satisfaction of nurses and reduce workplace bullying with the objective of retaining nurses [2]. Nurses need a supportive work environment to cope with organizational job stressors and negative experiences from supervisors, colleagues, and patients [13,68]. Previous studies provide insights into the psychosocial work environment of nurses [1,69], such as occupational stress and well-being, relationships with supervisors, job content and working conditions, and workgroup friendliness and warmth [70]. Evidence suggests that nurses in hospitals experience high levels of job-related stress and emotional and cognitive detachment from work demands [71]. 

Inappropriate emotional labor within these organizations is not unusual and includes factors such as emotional blackmail by supervisors, colleagues, subordinates, patients, and relatives [19]. A friendly and warm sense of community plays an important role in recruiting and retaining talented nurses [70]. Positive interrelationships are essential for developing attractive organizations and communities. Organizations should assist nurses in developing mental and physical equality in working conditions via perceptions of organizational support in order to reduce emotional blackmail in the work environment. Thus, to enhance self-awareness of belonging toward the organization, healthcare organizations should form a flexible and autonomous mechanism to encourage employees to voice and maintain the relationships to coordinate and evaluate the appropriateness of healthcare processes.

Second, leadership and the workplace environment are critical factors in organizations [29]. Healthcare organizations must be professional, hierarchical systems in order to maintain their daily practices [13]. Managers must shape a coordinated institution and offer training initiatives to increase self-awareness, self-evaluation, and sensitivity that will help nurses regulate their emotions in order to enhance organizational identity. The hospital should formulate policies and establish a channel for nurses to voice to the organization, which will assist leaders in having open attitudes toward employees’ active engagement in workplace activities.

Third, nurses with higher emotional intelligence are more sensitive to the need to regulate their emotions [47]. Relationships with peers, including friendships and identification, in the workplace, provide vehicles to alleviate emotional states [19]. In this context, managers should not only enhance organizational identity, but they should value nurses with high emotional intelligence and develop channels to allow them to express their emotions. Organizations should arrange relevant learning programs to enhance nurses’ emotional intelligence and establish a higher level of supportive context for nurses to engage in the decision-making process in order to facilitate higher awareness of organizational identification and to stay at an organization.

Last but not least, Haavisto et al. [72] identified social skills as a major domain in training nurses. How to regulate nurses’ emotions should be one of the on-job training curriculums to help them deal with their working environments and to deliver suitable nursing care. Healthcare organizations must shape a coordinated institution and offer training initiatives to increase nurses’ ability and attitude to enhance their emotional intelligence and control the hospital’s rules and procedures according to the requirements of the situation.

### 4.4. Limitations and Future Research

Several limitations should be recognized for future research. First, we collected cross-sectional, questionnaire-driven data. Future research can use longitudinal and in-depth contextual facets to design and gather multiple sources to gain a better understanding of relational factors and causality. 

Second, the concept and survey used in this study are eastern-based. Results may be diverse and varied due to the impact of different cultures. Chinese or eastern-based societies are considered collective societies [66]. Employees within this cultural situation will have a tendency to sacrifice their personal interests in order to achieve organizational goals [18]. Future research could focus on cross-cultural comparisons to verify these results. 

Third, our participants were the nurses of a public regional hospital in an urban area. Different organizational cultures and resources of hospitals could vary the relevant results. Researchers should extend different facets to examine the hypothesized relationships in different backgrounds of hospitals, such as different organizational cultures, private or public hospitals, the scale of hospitals, and the location. 

Fourth, past studies demonstrated that turnover intentions are negatively associated with experience [73]. Nurses from younger generations experience their work conditions as less consistent with their personal values and exhibit more burnout [29]. Therefore, demographic variables, such as differences between generations and departments of nurses, could be examined to analyze influential determinants of the hypothesized framework.

Fifth, individuals are not machines, and their reactions and decision-making processes are influenced by emotional issues, such as emotional intelligence [24]. Individuals with higher emotional intelligence will have a higher ability to dominate their interactions with others [24]. Past research has indicated that men seem to have a privileged position at work which is stereotyped as feminine in the healthcare institute [66]. Gender will affect the emotion, job satisfaction, and turnover intention of hospital nurses [74]. Future research could integrate gender and emotion theories and research to further illustrate the emotion-specific processes in healthcare systems.

Finally, our study only examined the effects of emotional blackmail on outcomes and variables, as well as the moderating effect of emotional intelligence between emotional blackmail and job satisfaction and turnover intentions. One avenue for future research could extend our study to enrich relevant fields by examining the antecedents of emotional blackmail and potential mediators, or moderators, of the framework, which could include organizational culture, organizational climate, leadership, power distance, and perceived alternative employment opportunities.

### 4.5. Conclusions

Nurses are valuable assets for healthcare systems. The demand for nurses is becoming a major challenge in healthcare facilities [5]. However, more and more patients view the nursing profession as a kind of service. In addition to catering to the many whims of patients and the restrictions of a professionally hierarchical organization, nurses must cope with unreasonable demands made by supervisors and colleagues in the stressful environment of healthcare organizations, all of which often exert an adverse effect on nurses. Emotions are vital to the nursing profession, and these have an influence on patient care decisions [75]. This study bridges the research gap to investigate the relationships among emotional blackmail, emotional intelligence, job satisfaction, and turnover intentions of nurses. The results revealed the direct relationships between emotional blackmail, job satisfaction, and turnover intentions, and job satisfaction partially mediated the relationship between emotional blackmail and turnover intentions. However, there is no statistical support that emotional intelligence moderates the relationships between emotional blackmail and job satisfaction and turnover intentions. Machová et al. [76] indicate motivated employee is a critical key to maintaining customers’ satisfaction and loyalty. While nurses suffer stress, burnout, or fatigue, they could become insensitive to patients’ needs [77]. These stresses could be detrimental to patient care and could lead to further psychological and physical issues for nurses [77,78]. If they are to embrace this dilemma, healthcare organizations must take the initiative and form strategies that will help balance nurses’ work stress. These strategies should aim to reduce unnecessary demands from patients and co-workers as well as in the socio-emotional domain. 

## Figures and Tables

**Table 1:** Summary of the hypotheses.

Hypothesis 1 (H1)	Emotional blackmail is positively associated with turnover intentions.
Hypothesis 2 (H2)	Emotional blackmail is negatively associated with job satisfaction.
Hypothesis 3 (H3)	Job satisfaction is negatively associated with turnover intentions.
Hypothesis 4 (H4)	Job satisfaction mediates the relationship between emotional blackmail and turnover intentions.
Hypothesis 5 (H5)	Emotional intelligence moderates the relationship between emotional blackmail and turnover intentions.
Hypothesis 6 (H6)	Emotional intelligence moderates the relationship between emotional blackmail and job satisfaction.
Hypothesis 7 (H7)	The indirect effect of emotional blackmail on turnover intentions through job satisfaction moderated by emotional intelligence.

**Table 2:** Summary of the participant profile.

Variables	Category	No. of Samples	Percentage (%)
Gender	Male	14	3.7
	Female	360	96.3
Age	20–29 years old	141	37.7
	30–39 years old	118	31.6
	40–49 years old	81	21.7
	50 years old (inclusive) and above	34	9.1
Education	High/vocational school	101	27.0
	4/2 year college	65	17.4
	University	197	52.7
	Master’s degree or above	11	2.9
Tenure	Less than 1 year	23	6.1
	1 year–less than 3 years	56	15.0
	3 years–less than 5 years	58	15.5
	5 years–less than 7 years	37	9.9
	7 years–less than 9 years	30	8.0
	More than 9 years	170	45.5
Division	Internal	195	52.1
	Surgical	74	19.8
	Outpatient	19	5.1
	Emergency	86	23.0
Level	N0	61	16.3
	N1	83	22.2
	N2	143	38.2
	N3	51	13.6
	N4	36	9.6

*n* = 374.

**Table 3:** Means, standard deviations, CR, AVE, and correlations among variables.

Variables	Mean	S.D.	CR	AVE	1	2	3	4
1. Emotional blackmail	3.017	1.080	0.969	0.570	**0.755**			
2. Emotional intelligence	5.432	0.688	0.972	0.690	−0.286 **	**0.831**		
3. Job satisfaction	5.134	1.074	0.949	0.860	−0.364 **	0.463 **	**0.927**	
4. Turnover intentions	3.216	1.461	0.947	0.856	−0.441 **	−0.287 **	−0.524 **	**0.925**

Notes: *n* = 374. S.D.: standard deviations; CR: composite reliability; AVE: average variance extracted. ** *p* < 0.01. The bold diagonal line shows the square root of AVE.

**Table 4:** Discriminant Validity: Heterotrait-monotrait (HTMT) criterion.

Variables	Emotional Blackmail	Emotional Intelligence	Job Satisfaction	Turnover Intentions
Emotional blackmail	**-**			
Emotional intelligence	0.308	**-**		
Job satisfaction	0.376	0.507	**-**	
Turnover intentions	0.467	0.315	0.568	**-**

Notes: *n* = 374. The threshold of HTMT should be below 0.85.

**Table 5:** The results of regression analysis.

	Turnover Intentions			Job Satisfaction	
	Model 1	Model 2	Model 3	Model 4	Model 5
Gender	00.069	00.048	00.017	−0.095	−0.079
Age	−0.097	−0.194 **	−0.122 *	0.106	0.182 *
Education	−0.006	−0.047	−0.051	−0.042	−0.011
Tenure	−0.123	−0.121	−0.139 *	−0.043	−0.045
Division	0.089	0.062	0.013	−0.143 **	−0.122 *
Level	0.074	0.084	0.076	−0.012	−0.020
EB		0.488 ***	0.337 ***		−0.379 ***
JS			−0.398 ***		
R^2^	0.031	0.256	0.388	0.030	0.165
△R^2^	0.031	0.225	0.132	0.030	0.136
*F*	10.971	1100.590 ***	780.679 ***	10.889	590.434 ***

Notes. *n* = 374. * *p* < 0.05, ** *p* < 0.01, and *** *p* < 0.001. EB: Emotional Blackmail; JS: Job Satisfaction.

**Table 6:** Bootstrap mediation, moderation, and moderated mediation effects.

	Estimate	BootSE	BootLLCI	BootULCI
mediation effect				
EB→JS→TI	0.1478	0.0388	0.0828	0.2354
moderation effect				
EB*EI→TI	0.2281	0.0810	0.0688	0.3873
EB*EI→JS	0.1019	0.0630	−0.0489	0.2268
moderated mediation				
EB*EI→JS→TI	−0.0589	0.0418	−0.1347	0.0292

Notes. *n* = 374. EB: Emotional Blackmail; EI: Emotional Intelligence; TI: Turnover Intention; JS: Job Satisfaction.

**Table 7:** The results of the hypotheses.

Hypothesis	Results
H1: Emotional blackmail is positively associated with turnover intentions.	Supported
H2: Emotional blackmail is negatively associated with job satisfaction.	Supported
H3: Job satisfaction is negatively associated with turnover intentions.	Supported
H4: Job satisfaction mediates the relationship between emotional blackmail and turnover intentions.	Supported
H5: Emotional intelligence moderates the relationship between emotional blackmail and turnover intentions.	Not supported
H6: Emotional intelligence moderates the relationship between emotional blackmail and job satisfaction.	Not supported
H7: The indirect effect of emotional blackmail on turnover intentions through job satisfaction moderated by emotional intelligence.	Not supported

## Data Availability

The data used to support the findings of this study are available from the corresponding author by request.

## Appendix A

	**Indicator**	**Source**
Emotional blackmail	1. The supervisor will use the threat of high- or low-performance appraisal to make performance requirements. 2. The supervisor would humiliate me with inappropriate words. 3. The supervisor would put the onus on me to get what he/she wanted. 4. The supervisors would privately accuse me of failings. 5. The supervisor would threaten me with consequences if I didn’t follow orders. 6. I would feel guilty when my supervisor said that the reason the job didn’t work out was because I didn’t have help. 7. If I fail to meet my supervisor’s demands, I feel guilty. 8. The supervisor would complain that I was selfish and didn’t think about the hospital. 9. The supervisor is too concerned about my work situation, which increases my pressure and affects work efficiency. 10. Colleagues would ask me to be on their side or else they would sideline me. 11. Colleagues would privately criticize my mistakes. 12. Colleagues will blame me for doing something wrong. 13. I feel guilty when co-workers say that work cannot be done because of my absence. 14. Colleagues are more capable than I am and make me feel guilty when I can’t help. 15. Colleagues will use the mourning strategy to gain my sympathy to achieve their goals. 16. Colleagues often present alternative choices, for example: choose to help me or help him/her. 17. Colleagues will give me special treatment in order to obtain my assistance. 18. Colleagues would openly complain that I was selfish and unwilling to help. 19. When I have a problem at work, I feel embarrassed that my colleagues are overly concerned. 20. Patients or their families would humiliate me with inappropriate language for dissatisfaction with the service. 21. Patients or their families have threatened me to speak to the supervisor because they are not satisfied with the service process. 22. Patients or their family members threatened me to complain to the media or public opinion representatives because they were not satisfied with the service process. 23. Patients or their families will spread false rumors because they are dissatisfied with the service results. 24. Patients or their families will say that they know the senior management of the hospital and ask me to provide better service. 25. When I was new to the business, it was stressful for patients or their families to replace scolding with empathy.	Liu and Jhuang, 2016 [51]
Emotional intelligence	1. I have a good sense of why I have certain feelings most of the time. 2. I have good understanding of my own emotions. 3. I really understand what I feel. 4. I always know whether or not I am happy. 5. I always know my friends’ emotions from their behavior. 6. I am a good observer of others’ emotions. 7. I am sensitive to the feelings and emotions of others. 8. I have good understanding of the emotions of people around me. 9. I always set goals for myself and then try my best to achieve them. 10. I always tell myself I am a competent person. 11. I am a self-motivating person. 12. I would always encourage myself to try my best. 13. I am able to control my temper so that I can handle difficulties rationally. 14. I am quite capable of controlling my own emotions. 15. I can always calm down quickly when I am very angry. 16. I have good control of my own emotions.	Law et al., 2004 [44]
Job satisfaction	1. Overall, I like working here. 2. Overall, I am satisfied with my job. 3. Overall, I don’t like my job.	Cammann et al., 1979 [53]
Turnover intention	1. I am seriously considering quitting my current job. 2. I’m seriously looking for other job opportunities. 3. I expect to quit my current job within a year.	Kelloway et al., 1999 [54]
